# Comparative Study of Blood Loss and Hemostasis in Doppler-Guided Hemorrhoidal Artery Ligation Versus Open Hemorrhoidectomy

**DOI:** 10.7759/cureus.102186

**Published:** 2026-01-24

**Authors:** Muhammad Usama Talib, Faiza Hameed, Qambar A Laghari, Zameer Hussain Laghari, Aijaz Ahmed Shaikh, Renad Al Mefleh

**Affiliations:** 1 General Surgery, University Hospitals of Morecambe Bay NHS Foundation Trust, Kendal, GBR; 2 General Surgery, Liaquat University of Medical and Health Sciences, Jamshoro, PAK; 3 Surgery, Liaquat University of Medical and Health Sciences, Jamshoro, PAK; 4 Pediatrics, Jordanian Royal Medical Services, Amman, JOR

**Keywords:** hemorrhoidal disease, intraoperative bleeding, minimally invasive surgery, postoperative hemorrhage, surgical outcomes

## Abstract

Background

Hemorrhoidal disease is among the most commonly seen anorectal disorders. Surgical treatment is routinely indicated in advanced stages or when conservative measures fail. Although conventional open hemorrhoidectomy remains an effective procedure, it is often accompanied by considerable intraoperative bleeding and postoperative hemorrhagic complications. Doppler-guided hemorrhoidal artery ligation (DGHAL) is a minimally invasive approach that targets the arterial supply of hemorrhoidal tissue and has been proposed to improve hemostatic control. This study aimed to compare intraoperative blood loss and bleeding-related outcomes between DGHAL and open hemorrhoidectomy.

Methods

This prospective, observational, non-randomized comparative study was conducted from March 2023 to February 2025 at Liaquat University of Medical and Health Sciences, Jamshoro, Pakistan. Adults aged 18-65 years with grade II-IV hemorrhoids were enrolled using non-probability consecutive sampling. Grade II cases were included only after documented failure of conservative therapy or rubber band ligation, or in the presence of recurrent bleeding, symptomatic prolapse, or hemorrhoid-related anemia. Patients underwent Doppler-guided hemorrhoidal artery ligation (DGHAL) or open hemorrhoidectomy according to predefined institutional protocols; allocation was non-random and based on disease severity, prolapse extent, bleeding severity, patient preference, and Doppler availability. Intraoperative blood loss was measured using suction volume and sponge weight difference. Additional hemostatic intervention was defined as persistent bleeding lasting >30 seconds or visible arterial spurting requiring sutures or electrocautery. Clinically significant postoperative bleeding was recorded within 24 hours and 14 days. Data were analyzed using SPSS version 26 (IBM Corp., Armonk, New York, USA) with multivariable regression adjustment.

Results

A total of 140 patients were analyzed, with 70 individuals in each treatment group. Demographic and baseline clinical characteristics were similar between groups. The mean intraoperative blood loss was significantly lower in patients undergoing DGHAL compared with those treated by open hemorrhoidectomy (32.4 ± 14.8 mL vs 68.9 ± 22.6 mL; p < 0.001). Patients in the DGHAL group required fewer supplementary hemostatic interventions and had significantly shorter operative times (p < 0.001). Both early and late postoperative bleeding events occurred less frequently following DGHAL, and the reduction in postoperative hemoglobin levels was also significantly smaller (p < 0.001). Multivariable analysis identified the type of surgical procedure as the most significant independent determinant of blood loss.

Conclusion

Doppler-guided hemorrhoidal artery ligation was associated with significantly lower intraoperative blood loss and improved short-term hemostatic outcomes compared to open hemorrhoidectomy. These findings support DGHAL as an effective alternative for achieving better perioperative bleeding control; however, they reflect short-term hemostatic advantages rather than overall procedural superiority.

## Introduction

Hemorrhoidal disease is among the most common anorectal conditions observed in surgical settings. It affects a substantial proportion of the adult population globally [1]. It is often diagnosed by symptomatic increase and distal dislocation of the normal cushions of anus. It leads to complications that include bleeding, prolapse, pain, pruritus, and mucus discharge [2]. Although hemorrhoids are not life-threatening, their influence on the quality of life and productivity in daily routine work is considerable.

The pathophysiology of hemorrhoids is multifactorial and involves both mechanical deterioration of the supportive tissues of these anal cushions and vascular hyperplasia with increased arterial inflow through arteriovenous shunts [3]. Surgical intervention is usually preserved for individuals with grade III and IV hemorrhoids or those with consistent indications despite being treated conservatively [1]. Conventional excisional hemorrhoidectomy, including the Milligan-Morgan technique, has long been regarded as the gold standard surgical treatment because of its effectiveness and low recurrence rates [2]. However, this procedure is associated with considerable postoperative pain, bleeding, delayed wound healing, and other complications, such as infection, stenosis, and incontinence [3,4].

In an effort to reduce surgical trauma and postoperative morbidity, minimally invasive techniques have been developed. Doppler-guided hemorrhoidal artery ligation (DGHAL), first described by Morinaga et al., works on the principle of identifying and ligation of the terminal branches of the superior rectal artery using a Doppler probe, thereby reducing arterial blood flow to the hemorrhoidal plexus [5]. This technique preserves the anoderm and rectal mucosa and aims to control symptoms by inducing the shrinkage of the hemorrhoidal cushions. Several studies have reported that DGHAL is associated with less postoperative pain, faster recovery, and fewer complications compared to conventional hemorrhoidectomy [6,7].

While most published studies comparing DGHAL with open hemorrhoidectomy have focused primarily on postoperative pain, hospital stay, and functional outcomes, limited data are available regarding the direct comparison of intraoperative blood loss and hemostatic efficacy between the two techniques. Bleeding remains a clinically important outcome, as excessive intraoperative or postoperative hemorrhage can increase morbidity, prolong recovery, and necessitate re-intervention [8]. Understanding the comparative hemostatic performance of these procedures is therefore essential for informed surgical decision-making. The objective of this study was to evaluate and compare intraoperative blood loss, hemostatic requirements, and postoperative bleeding outcomes between these two surgical techniques in patients with grade II to IV hemorrhoidal disease.

## Materials and methods

Current prospective observational comparative study was conducted in accordance with the Strengthening the Reporting of Observational Studies in Epidemiology (STROBE) guidelines. The research was carried out at Liaquat University of Medical and Health Sciences, Jamshoro, Pakistan. The study period spanned two years, from March 2023 to February 2025.

The study population comprised adult patients diagnosed with symptomatic grade II to IV hemorrhoidal disease who were planned for surgical management after failure of conservative treatment. Patients were allocated into two groups based on the surgical procedure selected by the treating consultant: DGHAL and open hemorrhoidectomy (Milligan-Morgan technique). As this was an observational study, no randomization was performed, and treatment allocation reflected routine clinical practice.

The sample size was calculated using OpenEpi software (Dean, Sullivan, Soe, Emory University, Rollins School of Public Health, Atlanta, GA, USA) for comparison of the mean intraoperative blood loss between two independent groups. Based on previously published literature indicating a mean difference of approximately 20 mL in blood loss between DGHAL and open hemorrhoidectomy, with a standard deviation of 35 mL, a power of 80%, and a two-sided alpha of 0.05, the minimum required sample size was calculated to be 60 patients per group [9,10]. To account for potential dropouts and incomplete data, a total of 140 patients were enrolled, with 70 patients in each group.

Non-probability consecutive sampling was employed. All eligible patients presenting to the participating centers during the study period and meeting the inclusion criteria were invited to participate. Inclusion criteria were patients aged 18 to 65 years, of either gender, diagnosed with grade II to IV hemorrhoids, and fit for surgery under regional or general anesthesia. Exclusion criteria included patients with bleeding diathesis, chronic liver disease, inflammatory bowel disease, colorectal malignancy, previous anorectal surgery, concurrent anal fissure or fistula requiring intervention, pregnancy, and those on long-term anticoagulant or antiplatelet therapy that could not be safely discontinued preoperatively.

Baseline demographic and clinical variables, including age, gender, body mass index, hemorrhoid grade, duration of symptoms, and comorbidities such as diabetes mellitus and hypertension, were recorded preoperatively. These variables were considered potential confounders due to their possible influence on intraoperative blood loss and postoperative hemostasis. To minimize confounding, surgeries were performed by consultant surgeons with at least five years of post-fellowship experience, and standardized perioperative protocols were followed at both centers.

Intraoperative blood loss was the primary outcome measure and was assessed by calculating the volume in the suction canister after subtracting the volume of irrigation fluid, along with the weight difference of surgical sponges measured before and after use, assuming a 1 g weight increase equivalent to 1 mL of blood. Hemostasis was evaluated intraoperatively by recording the need for additional sutures, electrocautery, or topical hemostatic measures, and postoperatively by documenting early postoperative bleeding within 24 hours and delayed bleeding within 14 days requiring medical or surgical intervention. Operative time was measured from skin incision to completion of wound dressing. Postoperative hemoglobin levels were assessed 24 hours after surgery to objectively evaluate blood loss.

All patients were followed during their hospital stay and subsequently in the outpatient clinic at two weeks postoperatively. Any postoperative complications related to bleeding were documented. Missing data were anticipated to be minimal due to the prospective design; however, if any variable had missing values of less than 5%, complete case analysis was performed. Patients with missing primary outcome data were excluded from the final analysis to avoid misclassification bias.

The Statistical Package for Social Sciences (SPSS) version 26 (IBM Corp., Armonk, New York, USA) was used to enter and analyze the data. The Shapiro-Wilk test was used to determine if continuous variables were normal. Non-normally distributed continuous data were displayed as median with interquartile range and compared using the Mann-Whitney U test, while regularly distributed continuous variables were reported as mean ± standard deviation and compared across groups using the independent samples t-test. The chi-square test or Fisher's exact test, depending on the situation, was used to assess categorical variables that were reported as frequencies and percentages. To account for possible variables influencing intraoperative blood loss, multivariable linear regression analysis was used. Statistical significance was defined as a p-value of less than 0.05.

Before the study began, the Institutional Research Ethics Committee of Liaquat University of Medical and Health Sciences, Jamshoro, granted ethical permission (Ref: LUMHS/REC/118, Dated: 17-02-2023). All subjects provided written informed permission after being told of the study's objectives, surgery choices, possible risks, and advantages. Throughout the study, patient anonymity was scrupulously upheld, and all procedures were carried out in compliance with the Declaration of Helsinki's guiding principles.

## Results

During the course of the trial, 140 patients were recruited, 70 of whom had open hemorrhoidectomy and 70 of whom underwent Doppler-guided hemorrhoidal artery ligation (DGHAL). Since there were no missing data for either the primary or secondary outcome variables, all recruited patients finished the trial and were included in the final analysis.

Table 1 summarizes the research population's baseline clinical and demographic characteristics. There was no statistically significant difference (p = 0.46) in the mean age of patients in the open hemorrhoidectomy group (45.9 ± 9.8 years) and the DGHAL group (44.6 ± 10.2 years). In both groups, the majority of patients were men. The two cohorts were sufficiently comparable at baseline, as seen by the lack of significant variations between the groups in terms of body mass index, hemorrhoid grade distribution, duration of symptoms, or incidence of comorbidities, such as diabetes mellitus and hypertension.

**Table 1:** Baseline demographic and clinical characteristics of the study population (n = 140). DGHAL: Doppler-guided hemorrhoidal artery ligation, IQR: interquartile range.

Variable	DGHAL (n = 70)	Open hemorrhoidectomy (n = 70)	Test statistic	p-value
Age (years, mean ± SD)	44.6 ± 10.2	45.9 ± 9.8	t = 0.74	0.460
Male gender, n (%)	46 (65.7)	44 (62.9)	χ² = 0.12	0.730
BMI (kg/m², mean ± SD)	26.8 ± 3.9	27.2 ± 4.1	t = 0.59	0.550
Grade II hemorrhoids, n (%)	22 (31.4)	20 (28.6)	χ² = 0.13	0.720
Grade III hemorrhoids, n (%)	30 (42.9)	32 (45.7)	χ² = 0.11	0.740
Grade IV hemorrhoids, n (%)	18 (25.7)	18 (25.7)	—	—
Duration of symptoms (months, median (IQR))	14 (9–20)	15 (10–22)	U = 2338	0.480
Diabetes mellitus, n (%)	18 (25.7)	20 (28.6)	χ² = 0.15	0.700
Hypertension, n (%)	21 (30.0)	23 (32.9)	χ² = 0.13	0.720

Intraoperative outcomes are presented in Table 2. Mean intraoperative blood loss was significantly lower in the DGHAL group (32.4 ± 14.8 mL) compared to the open hemorrhoidectomy group (68.9 ± 22.6 mL), and this difference was statistically significant (p < 0.001). The need for additional hemostatic measures was also significantly lower in the DGHAL group. Operative time was shorter in the DGHAL group, with a mean duration of 34.7 ± 7.9 minutes compared to 46.3 ± 9.2 minutes in the open hemorrhoidectomy group (p < 0.001).

**Table 2:** Intraoperative outcomes and hemostasis parameters. DGHAL: Doppler-guided hemorrhoidal artery ligation.

Parameter	DGHAL (n = 70)	Open hemorrhoidectomy (n = 70)	Test statistic	p-value
Intraoperative blood loss (mL, mean ± SD)	32.4 ± 14.8	68.9 ± 22.6	t = 11.1	<0.001
Operative time (minutes, mean ± SD)	34.7 ± 7.9	46.3 ± 9.2	t = 8.0	<0.001
Additional sutures required, n (%)	6 (8.6)	24 (34.3)	χ² = 14.1	<0.001
Use of electrocautery, n (%)	8 (11.4)	29 (41.4)	χ² = 16.0	<0.001

Postoperative bleeding outcomes and hemoglobin changes are detailed in Table 3. Early postoperative bleeding within 24 hours was observed in three patients (4.3%) in the DGHAL group compared to 11 patients (15.7%) in the open hemorrhoidectomy group (p = 0.028). Delayed postoperative bleeding within 14 days occurred significantly less frequently in the DGHAL group. The mean postoperative hemoglobin drop at 24 hours was significantly lower in the DGHAL group than in the open hemorrhoidectomy group (p < 0.001).

**Table 3:** Postoperative bleeding outcomes and hemoglobin changes. DGHAL: Doppler-guided hemorrhoidal artery ligation.

Outcome	DGHAL (n = 70)	Open hemorrhoidectomy (n = 70)	Test statistic	p-value
Early postoperative bleeding, n (%)	3 (4.3)	11 (15.7)	χ² = 4.84	0.028
Delayed bleeding (≤14 days), n (%)	2 (2.9)	9 (12.9)	χ² = 4.79	0.029
Postoperative Hb drop (g/dL, mean ± SD)	0.6 ± 0.3	1.4 ± 0.6	t = 9.5	<0.001
Re-intervention for bleeding, n (%)	1 (1.4)	6 (8.6)	Fisher’s exact	0.048

Multivariable linear regression analysis was performed to assess factors independently associated with intraoperative blood loss, adjusting for age, gender, body mass index, hemorrhoid grade, diabetes mellitus, and hypertension. As shown in Table 4, the type of surgical procedure remained the strongest independent predictor of blood loss, with open hemorrhoidectomy associated with a significantly higher blood loss compared to DGHAL (β = 34.6 mL, p < 0.001). No other covariates demonstrated a statistically significant independent association.

**Table 4:** Multivariable linear regression analysis for predictors of intraoperative blood loss. DGHAL: Doppler-guided hemorrhoidal artery ligation.

Variable	β coefficient (mL)	Standard error	95% CI	p-value
Open hemorrhoidectomy (vs DGHAL)	34.6	3.9	26.9–42.3	<0.001
Age (years)	0.12	0.09	−0.06–0.30	0.190
Gender, male	1.8	2.7	−3.5–7.1	0.510
BMI (kg/m²)	0.31	0.28	−0.24–0.86	0.270
Grade IV hemorrhoids	2.6	3.1	−3.5–8.7	0.400
Diabetes mellitus	1.9	2.8	−3.6–7.4	0.500

## Discussion

Demographic and clinical features at baseline were similar between both the groups, having not statistically (p≥0.05) significant differences among the groups in age, gender distribution, body mass index, hemorrhoid grade, duration of symptoms, or comorbidities, such as diabetes mellitus and hypertension. This comparability strengthens the internal validity of the study and suggests that the seen variances in outcomes are primarily attributable to the surgical technique rather than baseline patient-related confounders. Similar demographic homogeneity has been reported in previous comparative studies evaluating DGHAL and conventional hemorrhoidectomy, supporting the appropriateness of the comparison [7,11].

A key finding of this study was the significantly lower intraoperative blood loss observed in the DGHAL group. This can be explained by the targeted ligation of terminal branches of the superior rectal artery under Doppler guidance, which effectively reduces arterial inflow to the hemorrhoidal plexus without extensive tissue excision. In contrast, open hemorrhoidectomy involves excision of hemorrhoidal tissue with wide dissection of anoderm and mucosa, predisposing to greater bleeding. Khalil et al. reported a similar reduction in intraoperative blood loss with Doppler-guided hemorrhoidal artery ligation compared to conventional hemorrhoidectomy, attributing this benefit to precise arterial control and minimal tissue trauma [12]. Our findings are consistent with these observations and further reinforce the hemostatic advantage of DGHAL.

The reduced requirement for additional hemostatic measures, such as sutures and electrocautery, in the DGHAL group further supports the effectiveness of Doppler-guided arterial ligation in achieving intraoperative hemostasis. Excessive use of electrocautery in open hemorrhoidectomy, as observed in our study, has been associated with increased tissue damage and postoperative pain, as previously highlighted in the literature [6]. The lower reliance on adjunctive hemostatic techniques in DGHAL reflects a more controlled and physiologically oriented approach to hemorrhoidal surgery.

Operative time was also significantly shorter in the DGHAL group. Although DGHAL requires familiarity with Doppler equipment, once the learning curve is overcome, the procedure avoids extensive dissection and wound creation, leading to reduced operative duration. Similar reductions in operative time have been reported by Khalil et al. and other authors comparing DGHAL with conventional hemorrhoidectomy, particularly in experienced hands [12]. Shorter operative time may translate into reduced anesthesia exposure and improved operating room efficiency.

Postoperative bleeding outcomes further favored DGHAL, with significantly lower rates of both early and delayed postoperative bleeding. Early postoperative bleeding in open hemorrhoidectomy is often related to sloughing of ligated pedicles or inadequate intraoperative hemostasis, while delayed bleeding may occur due to secondary infection or tissue necrosis. By contrast, DGHAL preserves the anoderm and mucosa, reducing the risk of secondary hemorrhage. The lower postoperative hemoglobin drop observed in the DGHAL group in our study objectively corroborates these clinical findings. Similar reductions in postoperative bleeding and hemoglobin loss following DGHAL have been documented in previous studies, which emphasize its safety profile in terms of bleeding complications [12,13].

The multivariable regression analysis demonstrated that the type of surgical procedure was the only independent predictor of intraoperative blood loss, even after adjusting for potential confounders, such as age, sex, body mass index, hemorrhoid grade, and comorbidities. This finding underscores that the surgical technique itself plays a decisive role in determining blood loss, rather than patient-related factors. A previous study has similarly reported that demographic variables and common comorbidities do not significantly influence bleeding outcomes when modern, minimally invasive hemorrhoidal procedures are employed [14].

Overall, the findings of this study align closely with previously published literature comparing DGHAL and open hemorrhoidectomy, confirming that DGHAL offers superior hemostatic outcomes with less blood loss and fewer bleeding-related complications [15,16].

The findings of this study have important clinical implications for the surgical management of hemorrhoidal disease. Doppler-guided hemorrhoidal artery ligation demonstrated significantly lower intraoperative blood loss, better hemostatic control, and fewer postoperative bleeding complications compared to open hemorrhoidectomy. These advantages suggest that DGHAL may be particularly beneficial in patients at higher risk of bleeding or in settings where minimizing surgical trauma and postoperative morbidity is essential. The shorter operative time and reduced need for additional hemostatic measures also support its use as a safe and efficient alternative to conventional hemorrhoidectomy in appropriately selected patients.

This study has certain limitations that should be acknowledged. First, the observational design without randomization may introduce selection bias, as the choice of surgical technique depended on surgeon preference and clinical judgment. Second, the study was conducted at two centers within a single city, which may limit the generalizability of the findings to other settings with different patient populations or surgical expertise. Third, long-term outcomes, such as recurrence, late complications, and functional results, were not assessed, as the primary focus was on blood loss and hemostatic parameters.

## Conclusions

Doppler-guided hemorrhoidal artery ligation is associated with significantly reduced blood loss, superior hemostasis, and lower postoperative bleeding rates compared with open hemorrhoidectomy in patients with grade II to IV hemorrhoids. Given its minimally invasive nature and favorable hemostatic profile, DGHAL represents an effective and safer surgical option for the management of hemorrhoidal disease. Further randomized controlled trials with longer follow-up are recommended to confirm these findings and to evaluate long-term outcomes.
